# Two-Level Micro-to-Nanoscale Hierarchical TiO_2_ Nanolayers on Titanium Surface

**DOI:** 10.3390/ma9121010

**Published:** 2016-12-13

**Authors:** Elena G. Zemtsova, Andrei Yu. Arbenin, Ruslan Z. Valiev, Evgeny V. Orekhov, Valentin G. Semenov, Vladimir M. Smirnov

**Affiliations:** Institute of Chemistry, Saint Petersburg State University, Universitetskii pr.26, St. Petersburg 198504, Russia; ezimtsova@yandex.ru (E.G.Z.); aua47@yandex.ru (A.Yu.A.); rzvaliev@gmail.com (R.Z.V.); zeka@list.ru (E.V.O.); val_sem@mail.ru (V.G.S.)

**Keywords:** sol-gel, dip coating, TiO_2_ nanolayers, shock drying, roughness, cracks, cell state, osteoblasts

## Abstract

Joint replacement is being actively developed within modern orthopedics. One novel material providing fast implantation is bioactive coatings. The synthesis of targeted nanocoatings on metallic nanotitanium surface is reported in this paper. TiO_2_-based micro- and nanocoatings were produced by sol-gel synthesis using dip-coating technology with subsequent fast (shock) drying in hot plate mode at 400 °C. As a result of shock drying, the two-level hierarchical TiO_2_ nanolayer on the nanotitanium was obtained. This two-level hierarchy includes nanorelief of porous xerogel and microrelief of the micron-sized “defect” network (a crack network). The thickness of TiO_2_ nanolayers was controlled by repeating dip-coating process the necessary number of times after the first layer deposition. The state of the MS3T3-E1 osteoblast cell line (young cells that form bone tissue) on the two-level hierarchical surface has been studied. Particularly, adhesion character, adhesion time and morphology have been studied. The reported results may serve the starting point for the development of novel bioactive coatings for bone and teeth implants.

## 1. Introduction

Metals covered by bioactive micro- and nanocoatings are advanced sources for the production of principal biomaterial types for modern dentistry, reconstructive surgery and orthopedics [1,2,3]. The increase of human life expectancy and life quality requires the development of materials for long term work in biological media. Metallic materials such as stainless steel, pure titanium and Ti alloys are widely used for production of implants for traumatology, orthopedics and dentistry. The most implants are fabricated from non-toxic titanium.

At the moment, traditionally used metallic materials have mostly reached their maximum tensile strength. That is why a novel nanostructuring approach is of great interest for the enhancement of the mechanical properties of metals and alloys. Nanostructuring can be realized e.g., via severe plastic deformation (SPD) [4].

In order to apply nanostructured materials for medical purposes, it is necessary to meet certain requirements [1]. The first is biocompatibility, i.e., the ability of a material to operate properly and not induce a negative action in the tissue. Material bioactivity is the other mandatory demand. It represents the ability to interact with body tissue.

Joint replacement is being actively developed within modern orthopedics. One novel important material providing fast implantation is bioactive coatings. Many researchers are investigating the possibility of modification of the surface layer by different compounds [5] and the production of coatings based on biogenic [6] and synthetic [7] polymers. However, not only the chemical composition of the coating but also its surface relief governs the bioactivity [8,9]. The cytological and histological analysis showed the effectiveness of coatings with two-level relief where the roughness is present on the nano- and microlevel [10].

Ti’s surface is not sufficiently bioactive. Bioactive nanocoating is required (usually, TiO_2_-based coatings are applied) [11,12]. The coated surface consists of film-like nanostructures of various roughness produced by chemical and physical approaches. Here, “roughness” can be defined as the set of micro- and nanosized “bumps” that form surface micro- and nanorelief. Oxide-based micro- and nanocoatings, including bioactive TiO_2_-based coatings, are usually fabricated using the following methods: anodic oxidation [13], plasma spraying [14], chemical deposition [15], sol-gel synthesis [16,17,18], by dip coating and spin coating [19,20], thermal sputtering [21], and direct current (DC) sputtering [22]. For the finest control of the layer thickness on the nanolevel, atomic layer deposition (ALD) technique is used [23,24,25,26,27]. ALD provides high precision and high homogeneity of layer thickness as well as nanoparticle diameter control on the substrates of any area [28,29].

According to [2,28,29], the presence of required three-dimensional roughness organization, on either the micro- or nanolevel, plays an important role in adsorption processes control on implant surface in biological media. Therefore, it is important to investigate new approaches for the modification of the surface with the roughness both on micro- and nanolevel. The sol-gel method in combination with dip coating technology on the flat substrate is one prospective way to produce samples with the required roughness type [16,17,18,30,31]. According to this technology, the wafer is taken out from the solution with the defined rate. It results in film with a controlled composition.

In this work, we studied the coating of anatase nanolayers onto a nanotitanium surface by means of sol-gel synthesis in dip coating mode. Anatase is more preferable for osteointegration than other phases of TiO_2_ [32] Resulting surface has two-level hierarchy of the structure. Further, the adhesion properties of the osteoblast cell line M3T3-E1 have been evaluated and the formation of a cell monolayer on experimental samples has been investigated. A two-level structure hierarchy of TiO_2_ micro- and nanolayer on nanotitanium is obtained for the first time using the shock drying technique in hot plate conditions (400 °C).

## 2. Materials and Methods

### 2.1. Materials

Reagents: titanium isopropoxide (IV) (Ti(OC_3_H_7_)_4_) 98+%, diethanolamine, isopropyl alcohol, distilled water, calcium acetate hydrate, and ammonium dihydrogen phosphate were purchased from Aldrich. Nanotitanium samples were prepared in “Nanomet” LLC, Ufa, Russia, from titanium Grade 4. Titanium rods of 1 m length were deformed on ECAP-Conform instrument at 400 °C and at the number of passes of 5. The value of accumulated true strain was 3.5. Detailed description of ECAP-C processing technique can be found in [33]. After ECAP-Conform processing, the billets were subjected to drawing at 200 °C resulting in production of rods with a diameter of 6 mm. The average grain size of nanotitanium was ca. 50–100 nm.

### 2.2. Methods

Surface topography was examined by scanning probe microscopy (Solver P47 Pro in the tapping mode on air) and scanning tunnel microscopy (Zeiss Supra 40VP, Carl Zeiss AG, Jena, Germany). The research was performed in the Interdisciplinary Research Center for Nanotechnology of Saint Petersburg State University (SPbSU). According to atomic force microscopy (AFM, NT-MDT, Zelenograd, Moscow, Russia), the surface of initial Ti substrate after mechanical and chemical treatment is characterized by a very low roughness level. The average height difference was ~3 nm. In the work, spectral ellipsometric complex “Ellips 1891 SAG” in the wave length range of 350–1000 nm was used. This equipment measures single- and multilayered surfaces on either flat or smooth substrate. Scanning electron microscopy (Zeiss Merlin, Carl Zeiss AG) was used to study structure and morphology of the layers. Elemental composition of the sample surface after TiO_2_ layer deposition was established by X-ray photoelectron spectroscopy (XPS, Thermo Fisher Scientific Escalab 250Xi, Thermo Fisher Scientific, Massachusetts, MA, USA). XPS provides analytical data only for surface layer with thickness less than 10 nm. The presence of organic components in the samples was controlled by IR-spectroscopy (IR Fourier spectrometer Nicolet 8700, Thermo Fisher Scientific).

### 2.3. TiO_2_ Gel Layer Formation on the Titanium Surface

**Figure 1:**
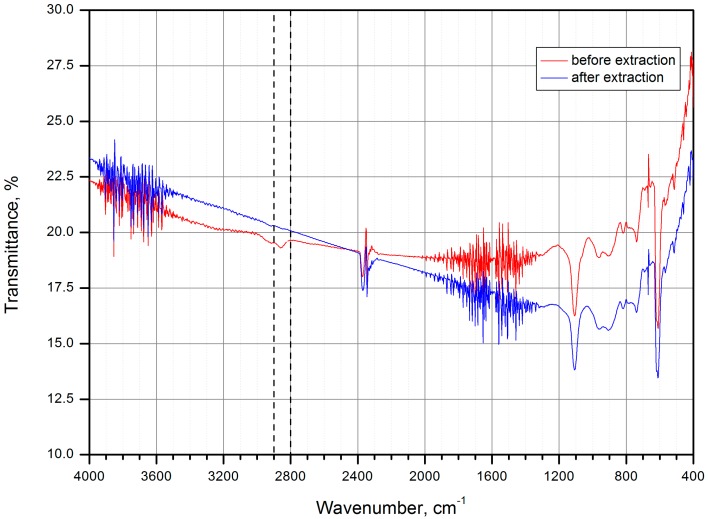
Infrared (IR) spectra of gel film before and after organic component removal.

The sol-gel method is quite a convenient technique for formation of continuous oxide gel layers (up to 100 nm) or continuous oxide gel films (from 100 nm to 10 μm) on solid substrates. Here oxide gel is a thermally treated oxide. Note that there are two methods for thickness control for such layers. The first approach is based on the variation of the rate of the wafer removal from the solution (mm/min). The second approach is based on dip coating repeated for the required number of times after the first layer deposition. Dip coating technique from non-aqueous solution was used for TiO_2_ layer deposition, as described in [31]. Continuous (non-defective) layer has been obtained by dip coating. The deposition of TiO_2_-based films was performed by using KSV NIMA Dip Coater Single Vessel, KSV NIMA, Biolin Scientific, Espoo, Finland. At first, titanium isopropoxide (TTIP) and diethanolamine (DEA) were dissolved in pure (water-free) isopropanol (i-PrOH). Then water was slowly added to the resulted solution under intensive stirring. The amount of water was two times more than titanium isopropoxide. This provides incomplete hydrolysis of titanium isopropoxide [34]. The final component ratio is TTIP/i-PrOH/DEA/H_2_O = 227/773/105/36 [35]. The solution was used for film deposition. Upon the deposition on a substrate, precipitate was transformed to the sol and then to the gel. In order to control the completeness of hydrolysis, the sample was placed into distilled water. Infrared (IR) spectra were obtained before and after extraction in order to confirm the absence of the organic component. Organic component removal was confirmed by the absence of C–H oscillation band at 2860 cm^−1^ in the sample after extraction comparing to the initial sample (see Figure 1). In order to obtain xerogel film, organic components were removed from the pores by extraction with boiling distilled water. The traces of water were removed by sample thermal treatment on air.

### 2.4. The Dip Coating Fabrication of Mesoporous Two-Level Micro- and Nanohierarchical TiO_2_ Layers on the Titanium Surface

The fabrication of micro- and nanolevel hierarchical TiO_2_ layers on Ti surface includes two main steps: (1) fabrication of the continuous (almost non-defective) TiO_2_ layers on metal surface; (2) shock drying in the hot plate conditions in order to design the required roughness via defects and cracks formation. For this purpose, the sample with the continuous layer, was placed on the preheated surface. That resulted in fast layer contraction because of liquid loss. The synthesis conditions leading to such defects will be discussed below.

#### 2.4.1. TiO_2_ Layers Fabrication on the Substrate Surface

Dip-coating technique was used due to its simplicity that allows fabricating the coatings with defined roughness. Ti substrate was fixed and placed into the solution. Then the substrate was removed from the solution with a rate of 100 mm/min. Near-surface hydrolysis of TTIP in the solution resulted in TiO_2_ film formation. Subsequently, the substrate was dried at 150 °C for 30 min in order to remove residual isopropyl alcohol. For the removal of residual organics, the extraction with water was performed, and thermal drying was repeated at 300 °C. In case of multilayered coating, to avoid the dissolution of titania layer, the wafer was dried at room temperature for 10 min after every removal from the solution. The last step was the same as for single layer coating.

#### 2.4.2. The Texturing of TiO_2_ Gel Layers on Titanium Surface by Fast (Shock) Drying

In this work, fast (shock) drying technique in hot plate conditions was applied for the first time. This approach enhances the roughness of TiO_2_ layer on nano-Ti by generation of various surface defects (board heating plate IKA HS7, IKA, Staufen, Germany. Titanium sample with applied TiO_2_ gel layer was placed on the heated surface. Film is contracted because of water loss. In some cases, when the stress relaxation at contraction was impossible, the defects, i.e., cracks, were formed in the film. When dip-coating finished, the wafers underwent shock drying at 400 °C for 10 min. Then the procedure was repeated in the same manner that is used in the Section 2.4, where residual organics was removed from wafers by boiling in water and then dried at 300 °C.

### 2.5. The Evaluation of Osteoblasts MC3T3-E1 Monolayer Formation on the Samples of TiO_2_-Coated Naotitanium

The evaluation of osteoblasts MC3T3-E1 cell adhesion properties and cell monolayer formation in the experimental samples was studied by scanning electron microscopy (SEM). The investigations were performed in the Institute of Cytology of the Russian Academy of Sciences (Saint Petersburg). All investigated samples were placed in Petri dishes and sterilized via ozonation. The application of cell line MC3T3-E1 suspension on the Ti surface was performed in a small volume of the culture medium so that the “drop” formed on the sample surface did not flow down. The cell concentration in the suspension was 1 × 10^5^ cm^−2^.

The samples with cell suspension thus applied on their surfaces were placed into CO_2_-incubator at +37 °C for 3 h. After that period of time, when the cell adhesion is finished, the nutrient solution was added into Petri dishes. For comparison and control, the cell suspension was also applied into dishes surface. After 5 days of cells cultivation, the nutrient solution was removed, washed three times by PBS buffer and diluted with 20-fold volume of 2.5% glutaraldehyde solution. The evaluation of cell condition (adhesion character and cell spreading on samples surface) was performed by SEM (JSM-35.7, Tokyo, Japan).

## 3. Results and Discussion

### 3.1. Thickness Regulation of TiO_2_ Gel Layer by Removal Rate Variation of the Nanotitanium Wafer from the Solution

**Figure 2:**
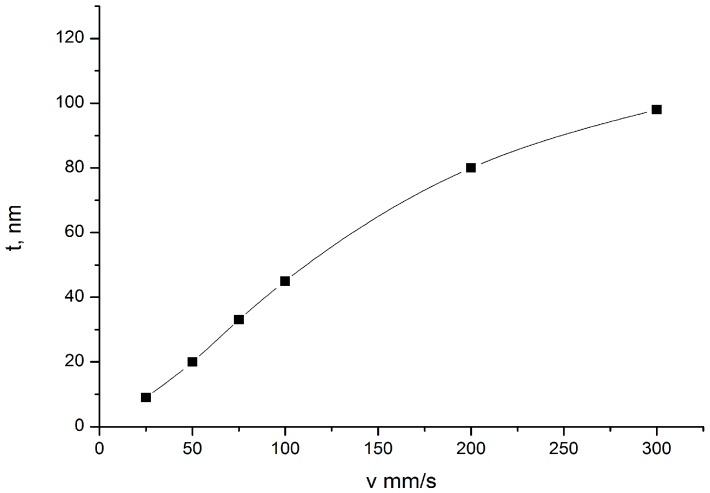
The effect of removal rate on TiO_2_ gel layer thickness.

The effect of removal rate of the nanotitanium wafer from the solution on TiO_2_ gel layer thickness was studied in the range of 25–300 mm/min. Linear dependence is observed within the range of 50–100 mm/min (Figure 2). At the rates higher than 100 mm/min diminished values were obtained, probably because at 100 mm/min full compensation of viscous drag by liquid cohesion is not possible. The rate of 75 mm/min was chosen for further investigations which is in the middle of linear dependence. That provides a guaranteed draining mechanism for film deposition.

**Figure 3:**
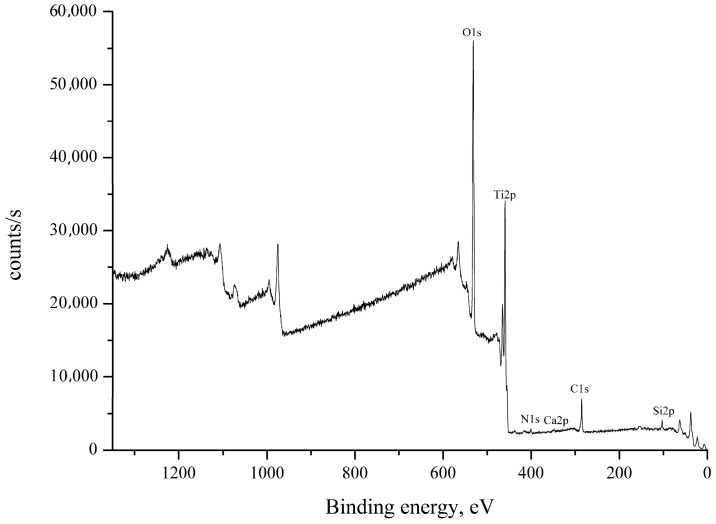
X-ray photoelectron spectrum of a nanotitanium sample with a TiO_2_ nanolayer with the thickness of 33 nm.

According to the results of the ellipsometric investigation, the thickness of the TiO_2_ layer on the sample obtained at the removal rate of 75 mm/min was 33 nm. Elemental analysis by XPS (see Figure 3 and Table 1) confirmed that the layer composition corresponds to TiO_2_.

**Figure 4:**
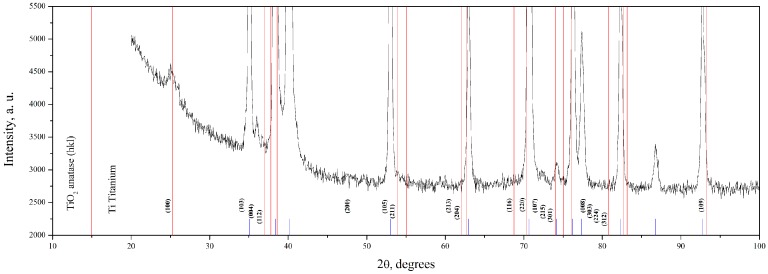
XRD analysis of 33 nm thick TiO_2_ nanolayer on nanotitanium (blue marks—titanium, red lines—anatase).

XRD analysis indicates that TiO_2_ film is in the anatase phase (Figure 4). Main peaks (marked blue) correspond to the titanium substrate; red lines with hkl indices indicate positions of anatase peaks.

### 3.2. Control of TiO_2_ Gel Layer Thickness Based on Repeating the Dip-Coating Process the Required Number of Times

Besides the removal rate variation, the layer thickness can also be increased by coating process cyclization. In this case, the deposition procedure differs from that described above. After the film deposition, the sample is protected from thermal and aqueous treatment in order to avoid the hydrolysis of reaction solution. After the removal, the sample underwent the dip-coating procedure again. Only after all the deposition cycles were finished, the sample was placed on the hot plate and then treated with the boiling water in order to extract the organic admixtures from the pores. Subsequently, the high temperature drying was used to obtain a xerogel layer. The analysis of the cyclization possibility was performed via the study of samples after 1–5 treatment cycles.

The linear dependence of film thickness on layers’ number was observed (Table 2). It should be noted that gels produced by sol-gel technology transform to xerogels during the purification from the organic molecules. Xerogels are porous 3D networks made of nanoparticles coupled to each other. The porosity of xerogels is primarily due to water removal from the initial gel. Taking into account the sol particle size of 1–100 nm, one should expect the nanoporosity of xerogel.

**Figure 5:**
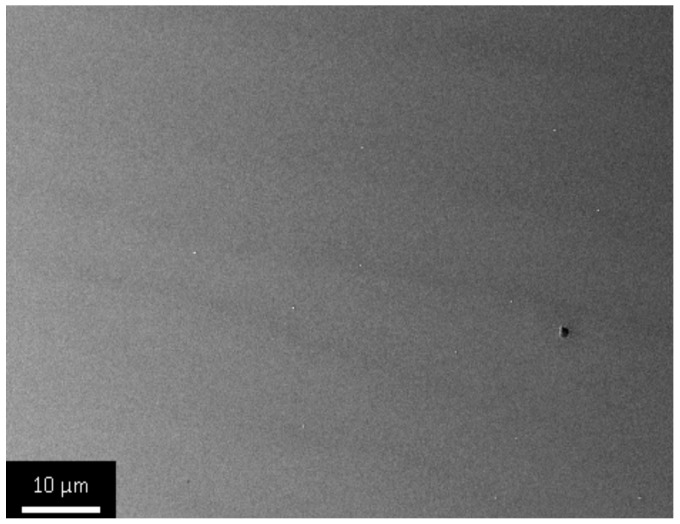
Low-resolution electron microphotograph of TiO_2_ gel layer deposited after one dip-coating cycle.

**Figure 6:**
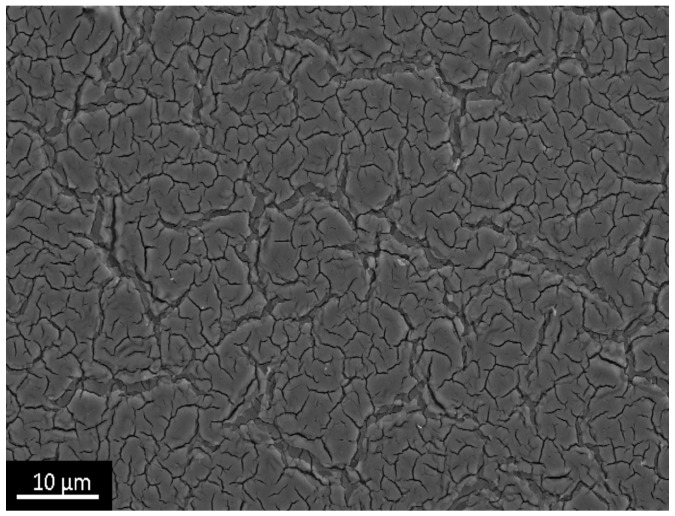
Low-resolution electron microphotograph of TiO_2_ gel layer deposited after five dip-coating cycles.

**Figure 7:**
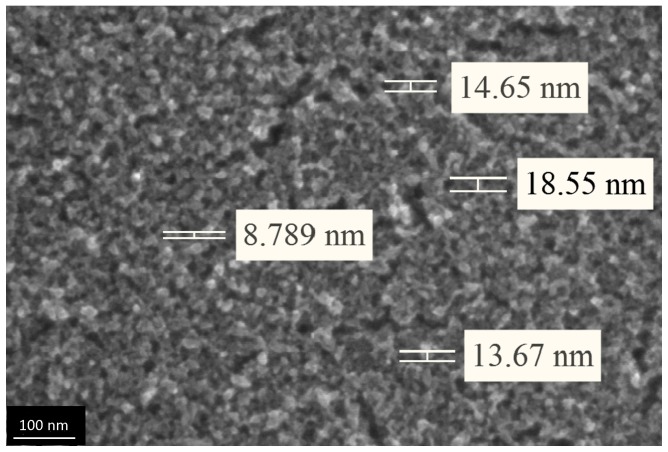
High resolution electron microphotograph of TiO_2_ gel layer deposited after one dip-coating cycle.

**Figure 8:**
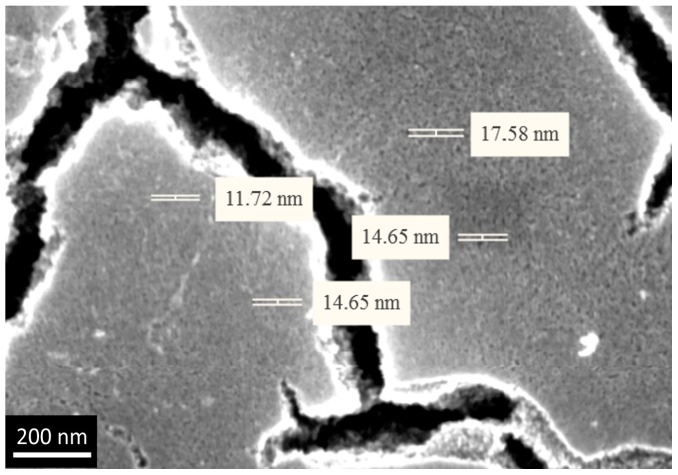
High-resolution electron microphotograph of TiO_2_ gel layer deposited after five dip-coating cycles.

We qualitatively evaluated porous structure of deposited TiO_2_ gel layer by means of electron microscopy. Even though this technique does not provide the information about bulk structure and porosity, this method of surface examination allows studying nanorelief formed by pore throats. The microscopy data showed that the sample surface after single treatment cycle is covered by almost continuous porous xerogel film (Figure 5). In the case of sample 5, after five treatment cycles, the TiO_2_ xerogel layer turns out to be covered by a dense network of micron-sized cracks (Figure 6). Here, the porous structure of the gel layer itself did not differ significantly from the one for sample 1. On the microphotograph of the sample after one treatment cycle, xerogel globules are observed. They are divided by pore throats with sizes in the range of 5–20 nm (Figure 7). The only difference of the sample 2 is presence of cracks. The sizes of pore throats lie in the same range (Figure 8).

This means the porous structure of the layer is not damaged upon crack growth, but the mechanical stability of the layer is decreased. It can be explained that during hot plate drying gel does not undergo significant contraction due to disperse medium loss and enhanced globules bonding. Undoubtedly, here the mechanical stresses appear. In the case of a thin layer, the relaxation takes place without a collapse. At the same time, the thick layers are relaxed by means of film fracture.

### 3.3. The Investigation of Morphology of TiO_2_ Layer, Obtained by Shock Drying

**Figure 9:**
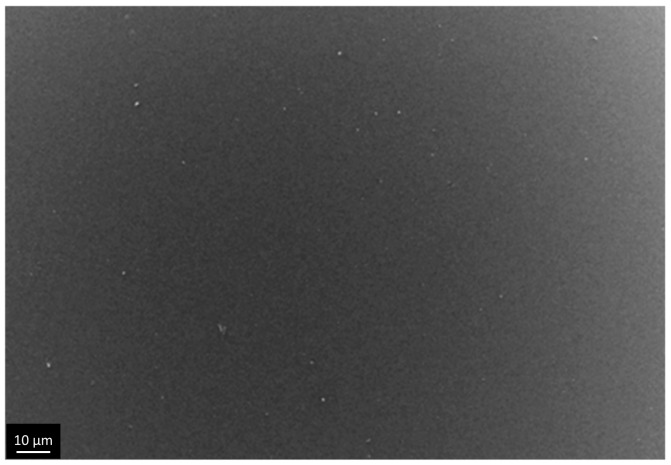
Microphotographs of TiO_2_ film with a thickness of 33 nm after shock drying, obtained at low resolution.

Initially, the effect of the film thickness on the defect arising during the shock drying was investigated. According to ellipsometric data, the initial dip-coating (removal rate of 75 mm/min) allowed to deposit the film with the thickness of 33 nm. The experiments on defect development on the sample surface with a thickness of 33 nm by shock drying technique were not successful. Even at a hot plate temperature of 400 °C, the defects in the film were quite rare and disordered. It should be noted that at these temperatures, the xerogel structure is preserved (Figure 9).

**Figure 10:**
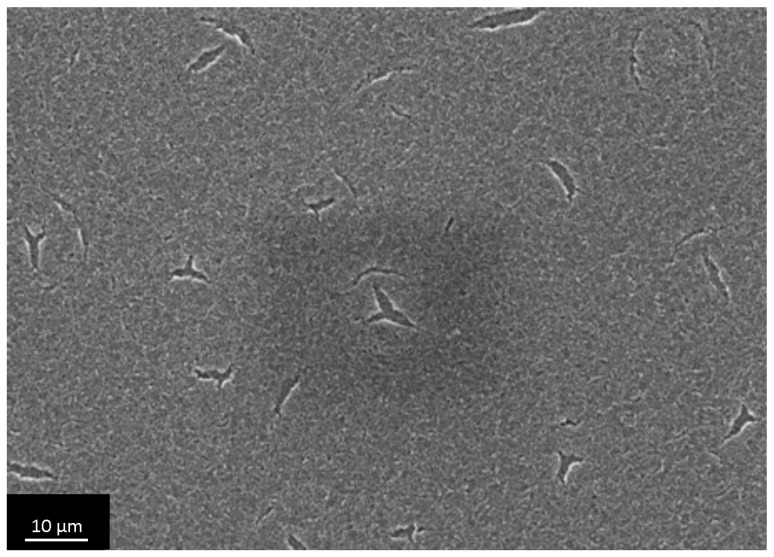
Microphotographs of TiO_2_ film with a thickness of 72 nm after shock drying, obtained at low resolution.

**Figure 11:**
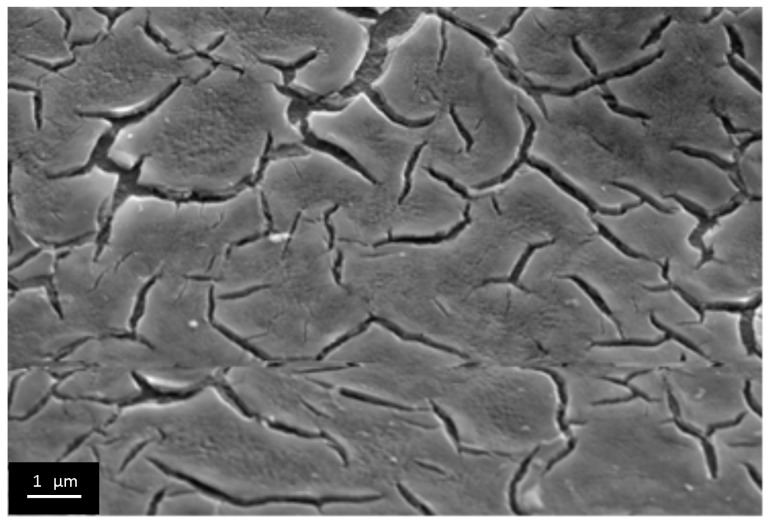
Microphotographs of TiO_2_ film with a thickness of 124 nm after shock drying, obtained at low resolution.

**Figure 12:**
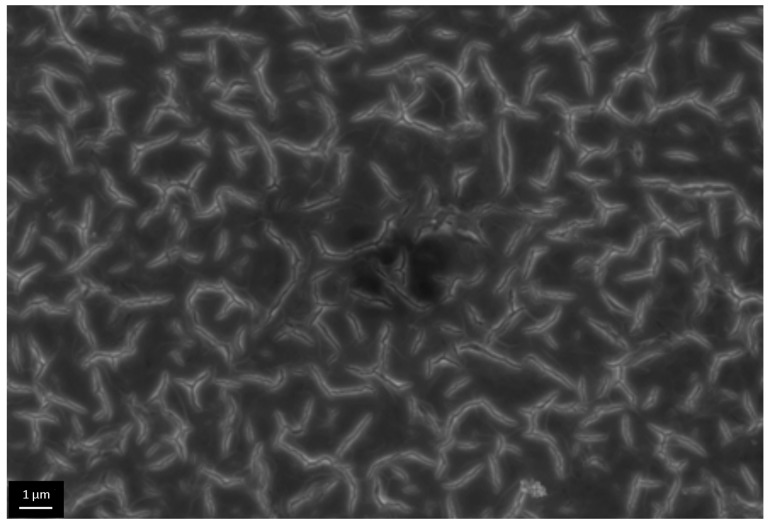
Microphotographs of TiO_2_ film with a thickness of 173 nm after shock drying, obtained at low resolution.

**Figure 13:**
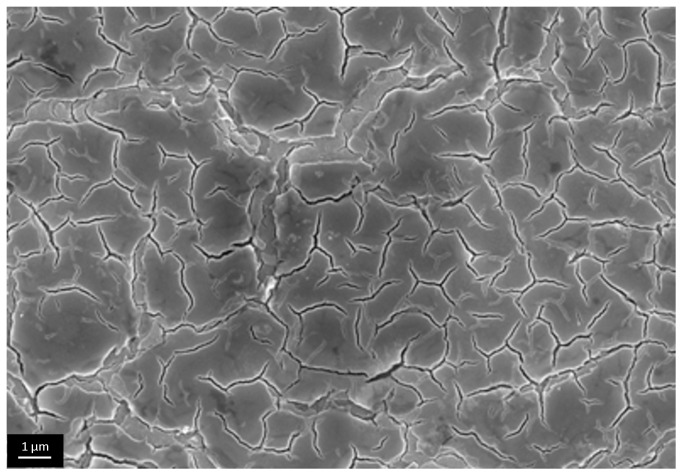
Microphotographs of TiO_2_ film with a thickness of 204 nm after shock drying, obtained at low resolution of the end face of the film.

**Figure 14:**
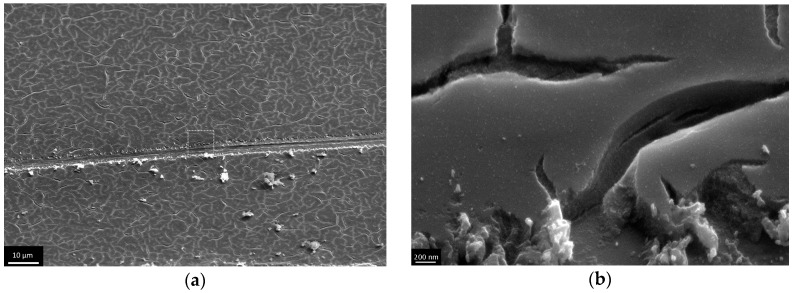
Microphotographs of the scratched TiO_2_ film with a thickness of 204 nm after shock drying, obtained at low resolution (**a**) and at high resolution (**b**).

The thicker films (72, 124, 173 and 204 nm) were obtained via dip coating process cyclization. As in the first experiment, these films underwent shock drying at 500 °C, which resulted in the ordering of different type on the micron level (Figure 10, Figure 11, Figure 12 and Figure 13). At a thickness of 72 nm, single defects appeared (Figure 10), and at a thickness of 124 nm and more, sparse cracks appeared (Figure 11 and Figure 12). When the film thickness reached 204 nm, a dense crack network was found (Figure 13). At first glance, it seems that cracks appeared layer by layer during every coating cycle as on the bottom of large cracks the small ones are observed. However, the detailed examination of the film scratched under the sharp angle indicates the presence of open cracks, penetrating the film until reaching the substrate material (Figure 14a). Also in high resolution microphotographs (Figure 14b) we can see the local exfoliation of TiO_2_ film, which suggests its layered structure.

Thus, shock drying leads to greater stress with increasing film thickness, which in turn at certain conditions allows crack-type defects to be obtained.

This can be explained as follows: during drying on a hot plate, the gel undergoes a significant contraction. Thus, the mechanical stability of the layer decreases with the increase of the layer thickness, medium evaporation and the enhancement of globule agglomeration. Undoubtedly, here the mechanical stresses appear. In case of a thin layer (less than 30 nm) relaxation can take place without collapse. In case of thicker layers (more than 30 nm) the relaxation proceeds by means of film disruption. To summarize, the use of dip-coating with subsequent shock drying provides nanolayers with two-level hierarchy of the structure. Nanorelief is developed by means of xerogel pore throats, whereas microrelief is due to the network of micron-sized cracks.

### 3.4. The Investigation of Osteoblasts Cell Line MC3T3-E1 State, i.e., Adhesion Character, Adhesion Time and Their Morphology on the Sample Surface

**Figure 15:**
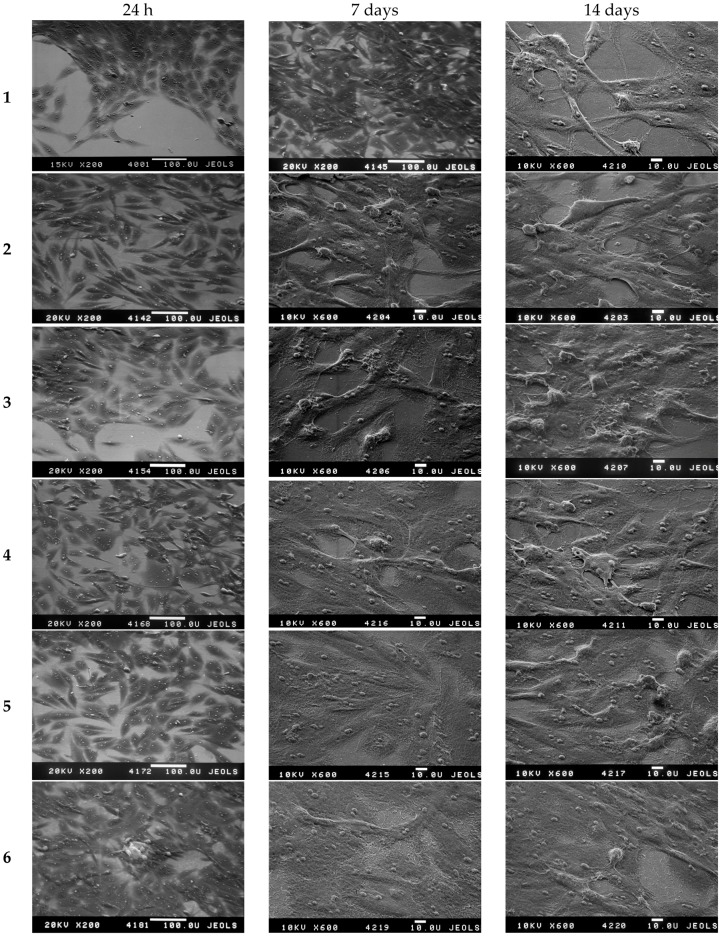
Characteristic SEM images for the samples **1**–**6** after adhesion and spreading of the human MC3T3-E1 cell line (**1**)—polished nanotitanium; (**2**)—sample with 33 nm TiO_2_ film; (**3**)—sample with 72 nm TiO_2_ film; (**4**)—sample with 124 nm TiO_2_ film; (**5**)—sample with 173 nm TiO_2_ film; (**6**)—sample with 204 nm TiO_2_ film.

In the present work, the effect of the structural characteristics of experimental samples 2–6 with two-level coating relief organization on cell adhesion was investigated. The osteoblast (young bone cells) cell line MC3T3-E1 (Figure 15) was used for analysis.

The investigation of the osteoblast MC3T3-E1 cell line on the surface of samples with oxide film (see Figure 15 samples 2–6) revealed that osteoblast adhesion is ≥85% after 72 h. These data indicate the absence of cytotoxicity of studied coatings and their applicability for implants. This is in accordance with the recent data obtained by other research groups [36,37].

It should be also mentioned that even for the sample after one cycle of oxide film deposition (thickness 33 nm), the formation of a continuous osteoblast monolayer on the surface with good adhesion is observed (Figure 15, sample 2). The sample with crack network (after five deposition cycles) showed not only high surface adhesion properties for the investigated cell line, but also a continuous cell layer (Figure 15, sample 6). This phenomenon indicates the ability of this surface to achieve accelerated osteosynthesis. That can be explained by the presence of two-level hierarchy of the structure formed by micron cracks and nanometer pores.

## 4. Conclusions

Fast (“shock”) drying of the TiO_2_ gel layer through sol-gel synthesis allows TiO_2_ nanolayers to be produced that have two-level hierarchical structures, i.e., nanorelief is obtained by means of pore throats of xerogel and microrelief is obtained via a micron “defect” network of various textures: sparse cracks and a crack network. In the case of a film with micron cracks, it was shown that the induction time of osteoblast differentiation is reduced via two-level hierarchy coating relief. That phenomenon indicates the ability of that surface to produce accelerated osteosynthesis. These structured materials could have potential applications for designing more complex biomaterials. The suggested technique is easy and reproducible.

## Figures and Tables

**Table 1:** XPS data of the sample with the thickness of 33 nm.

Name	Peak BE	Atomic %
**O1s**	530.81	63.03
**Ti2p**	459.27	31.57
**C1s**	285.07	2.52
**Si2p**	102.87	2.23
**N1s**	399.95	0.43
**Ca2p**	347.87	0.22

**Table 2:** Thickness increment during TiO_2_ gel layer cyclic deposition.

**The number of cycles**	1	2	3	4	5
**Film thickness (nm)**	33	72	124	173	204
**The thickness increment (nm)**	33	39	52	49	31
